# Prevalence and Patterns of Multimorbidity Among Rural Elderly: Findings of the AHSETS Study

**DOI:** 10.3389/fpubh.2020.582663

**Published:** 2020-11-05

**Authors:** Jaya Singh Kshatri, Subrata Kumar Palo, Trilochan Bhoi, Shakti Ranjan Barik, Sanghamitra Pati

**Affiliations:** Indian Council of Medical Research-Regional Medical Research Centre, Bhubaneswar, India

**Keywords:** multimorbidity, chronic diseases, older adults, cluster analysis, rural population

## Abstract

**Introduction:** In India, the proportion of older population is projected to increase from 8% in 2015 to 19% in 2050 and a third of the country's population will be older adults by end of the century. Multimorbidity is common among the elderly and the prevalence increases with age. Chronic conditions are most often present as clusters and it's critical to explore the prevalent pattern of clustering for better public health strategies.

**Method:** A cross-sectional study was conducted among 725 rural older adults (>60 years) in Tigiria block of Odisha, India. Multimorbidity status was assessed using the prior validated MAQ-PC tool. Survey was conducted using android tablets installed with open data kit software. While Euclidean distances using K-means clustering algorithm were used to estimate the similarity or dissimilarity of observations. The optimum numbers of clusters were determined using silhouette method. Data were analyzed using multiple open source packages of R statistical programming software ver-3.6.3.

**Result:** The overall prevalence of multimorbidity was 48.8% of which dyads (25%) were the most common form, followed by triads (15.2%). The prevalence of multimorbidity was higher in females (50.4%) than males (47.4%). The optimal number of clusters was found to be 3. While arthritis alone was a separate cluster, hypertension and acid peptic disease were in another cluster and all the rest conditions were included in the third cluster.

**Conclusion:** The cluster analysis to measure of proximity suggested arthritis, hypertension, and acid peptic disease are the diseases that occur mostly in isolation with the other chronic conditions in the rural elderly.

## Introduction

A disease is said to be chronic (or long term chronic condition) when it lasts for more than 1 year and needs ongoing health care. Feinstein in 1970 coined the term “Comorbidity” as any additional disorder that may exist or tend to occur during the clinical course of an index disease. In contrast, the term multimorbidity refers to a condition where there is co-occurrence of multiple chronic conditions without taking any of the disease as the index condition (1). Multimorbidity comprises of different conditions which may be concordant or discordant. However, some chronic conditions are more likely to cluster than others. This may be due to biological, behavioral, or environmental factors (2).

The prevalence of multimorbidity increases with age and is more common among the older adults. The overall prevalence of multimorbidity is reported between 24 and 83% in them (3). By 2050, the proportion of the world's population aged over 60 years is set to increase from 12% at present to 22% (4). Similarly, in India, the proportion of older population is projected to increase from 8% in 2015 to 19% in 2050 and a third of country's population will be older adults by end of the century (5).

Globally, the most common clustering pattern of multimorbidities is around depression, cardiometabolic disorders, and musculoskeletal disorders (2). In low, middle, and high income countries, the common clustering of multimorbidities shows a cardiorespiratory pattern such as angina, asthma, and COPD; a metabolic pattern such as diabetes, obesity, and hypertension; and a mental-articular pattern such as arthritis, and depression (6). In India most common cluster of multimorbidities are arthritis and hypertension followed by arthritis and cataract, and diabetes and hypertension (7, 8). Studies have shown that among the older adults, the prevalence of dyads (presence of two chronic conditions) is more compared to triads (presence of three chronic conditions) (31.8 vs. 15.5%) (9, 10).

An increasing trend of multimorbidity in the rural communities warrants an assessment of the burden in the rural older adults along with the patterns of chronic conditions in them. With this background, a comprehensive study was undertaken to assess the health status of the rural older adults using a syndemic approach Assessment of Health Status of the Elderly in Tigiria using Syndemic approach- the AHSETS study, taking into account multiple interrelated systems that contribute to heightened vulnerability within marginalized communities (11). We present the findings of the primary objective of this study which was to estimate the prevalence of multimorbidity among the rural older adults population in Tigiria block, Odisha, India, and explore the clustering and patterns of multiple chronic diseases among them.

## Methodology

### Study Design and Setting

The following study was Cross-sectional in design and carried out in the rural block of Tigiria in Cuttack district, Odisha, India, between June 2019 and February 2020. Tigiria is an administrative block of Odisha, India, consisting of 52 revenue villages with a total population of 74,639 as per Census 2011 (12). The study participants were residents of Tigiria block, Cuttack. We included those aged over 60 years who were conversant, comprehensible and provided their written informed consent to participate. We excluded seriously ill, bed ridden patients as well as those with severe cognitive impairment.

### Sample Size and Sampling

Assuming the prevalence of multimorbidity in the geriatric age group to be a conservative 20%, with 95% confidence level and width of confidence interval at 8%, beta of 0.20 and alpha of 0.05, the minimum sample size was calculated to be 407 (13). Assuming a design effect of 1.6 due to clustering and a non-response rate of 10%, the sample size required was rounded off to 725. Participants were selected using a cluster sampling technique from a list of 30 clusters (revenue villages) selected based on a Probability Proportional to Size (PPS) method. Systematic random sampling method was used in each of the clusters for identification of study households. All eligible participants from the selected household were recruited for the study. This was done until the necessary cluster size of 25 was attained. Immediate neighboring household was approached if the selected household failed to meet the eligibility criteria.

### Data Collection

Data were collected by trained field investigators using a pre tested tool based on Open Data Kit (ODK) software installed on android tablets. Multimorbidity status was assessed using the MAQ-PC tool, which was prior validated in the study population (14). Socio demographic data were collected following standard census of India operational definitions. Information on their personal habits such as smoking, chewing tobacco, and alcohol consumption behavior was collected.

### Quality Control

Data collection was commenced after a comprehensive training of the study staff using a standardized manual of operating procedures (MOP) for the study. Data were collected using tablets to reduce entry errors. Periodic verification of the data was done by the investigators by checking for its completeness, duplications, and range errors. Monitoring visits were carried out by the investigators weekly to review the data collection and protocol adherence. Existing validated tools for the Indian population were used after their translation (and back translation) into the regional language, Odia, to ensure generalizability.

### Statistical Analysis Plan

Data extraction, transformation, and cleaning were done using MS Excel. The data were scanned for outliers and no missing value was found. Frequencies and proportion were used as descriptive measures for categorical variables, with 95% confidence limits and mean with standard deviation for continuous variables. Data were checked for precision and bi-variate analysis was done using Chi-square test. Binary logistic regression models were used to adjust the odds for age and gender.

### Cluster Analysis

Euclidean distances were used as the distance measure to estimate the similarity or dissimilarity of observations. A K-means clustering algorithm by Hartigan et al. was used to divide the dataset into clusters (15). The optimum number of clusters was estimated using the average silhouette method, which assessed the quality of clustering (16). Multiple cluster plots with two dimensions and gender segregated dendrograms were built for visualization of distances and clusters. Analyses were done using multiple open source packages of R statistical programming software ver-3.6.3.

### Ethical Considerations

Ethical approval was obtained from the institutional human ethics committee of ICMR-RMRC Bhubaneswar (Approval No- ICMR-RMRCB/IHEC-2019/022). Written informed consent was obtained from all participants and the national ethical guidelines for biomedical research were followed (17).

## Results

**Figure 1:**
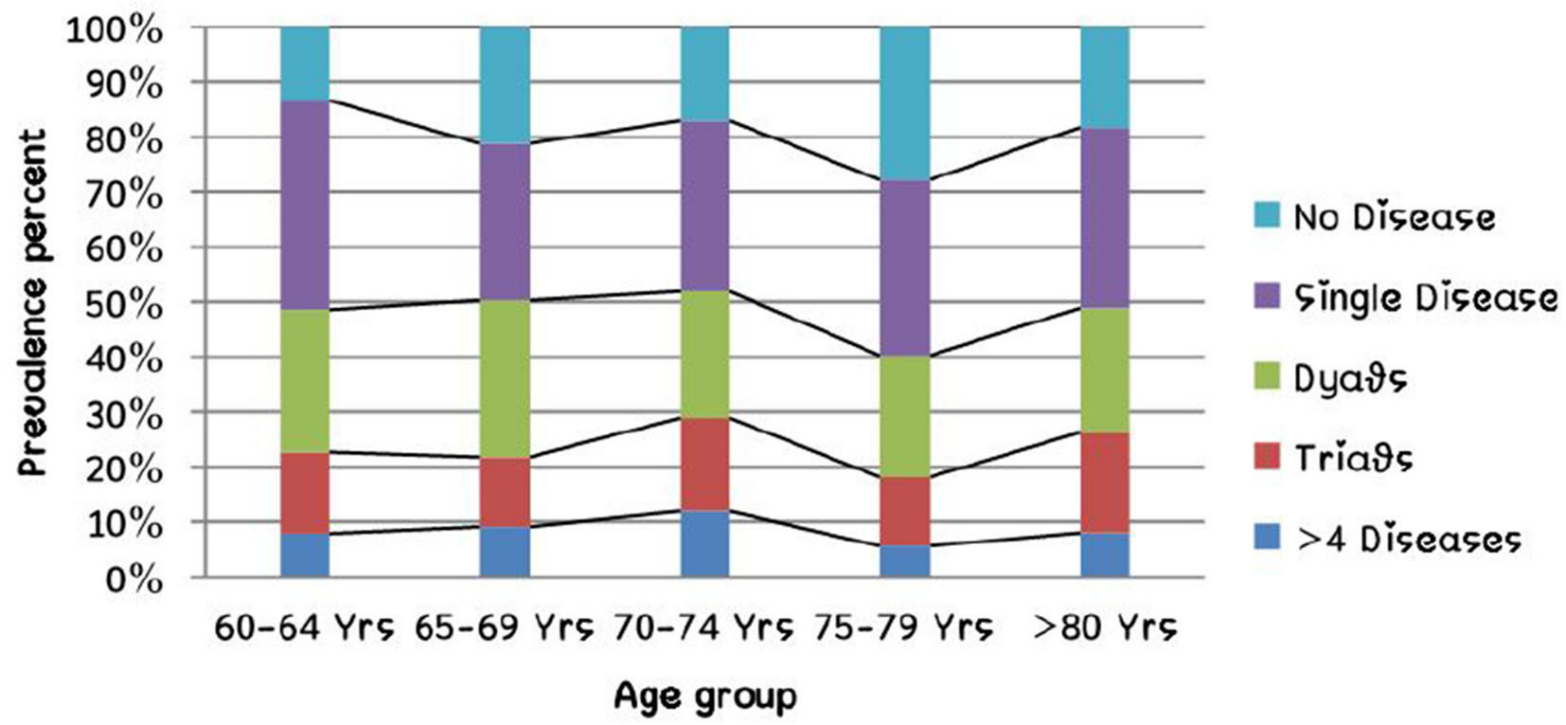
Relation of number of chronic disease and age group.

The study included a total of 725 rural older adults. Among them, 47.9% (*n* = 347) were female and the rest male. The age distribution of the participants was normal and the mean age was 70.24 years (*SD* = 8.37 years), and ranged between 60 and 106 years. The overall prevalence of multimorbidity was 48.8% (CI = 45.1–52.5%; *n* = 354) and among them dyads were most common (25%; CI: 21.8–28.2%) followed by triads (15.2%; CI: 12.6–17.9%). Four or more chronic diseases were seen in 63 persons (8.7%; CI: 6.7–10.9%). Among the study participants, 18.2% (*n* = 132; CI: 15.4–21.2%) had no chronic disease and 33.0% (*n* = 239; CI: 29.5–36.5%) had a single chronic disease. The socio-demographic characteristics of the study population are described in Table 1 and Figure 1 represents the relation between the number of chronic diseases and age group.

**Table 1:** Socio-demographic characteristics of the study population.

**Characteristic**		**Morbidity status**, ***n*** **(%)**			**Total**	**X^**2**^** **(*p*-value)**
		**No chronic disease**	**Single chronic disease**	**Multimorbidity**		
Age	60–69 Years	78 (16.8%)	144 (31.1%)	241 (52.1%)	463 (100%)	5.783 (0.448)
	70–79 Years	35 (20.5%)	63 (36.8%)	73 (42.7%)	171 (100%)	
	80–89 Years	17 (21.5%)	27 (34.2%)	35 (44.3%)	79 (100%)	
	≥90 Years	2 (16.7%)	5 (41.7%)	5 (41.7%)	12 (100%)	
Gender	Male	81 (21.4%)	118 (31.2%)	179 (47.4%)	378 (100%)	5.586 (0.061)
	Female	51 (14.7%)	121 (34.9%)	175 (50.4%)	347 (100%)	
Family type	Single	7 (13.2%)	23 (43.4%)	23 (43.4%)	53 (100%)	23.437 (0.001)*
	Nuclear	41 (16.0%)	85 (33.1%)	131 (51.0%)	257 (100%)	
	Joint	53 (16.7%)	95 (29.9%)	170 (53.5%)	318 (100%)	
	Extended	31 (32.0%)	36 (37.1%)	30 (30.9%)	97 (100%)	
Education	Illiterate	63 (18.2%)	126 (36.4%)	157 (45.4%)	346 (100%)	11.171 (0.083)
	Primary school	61 (20.3%)	91 (30.2%)	149 (49.5%)	301 (100%)	
	Secondary school	2 (5.3%)	13 (34.2%)	23 (60.5%)	38 (100%)	
	High school graduate or above	6 (15.0%)	9 (22.5%)	25 (62.5%)	40 (100%)	
Occupation	Not working	89 (15.7%)	184 (32.5%)	294 (51.9%)	567 (100%)	23.925 (<0.001)*
	Agriculture	34 (32.7%)	39 (37.5%)	31 (29.8%)	104 (100%)	
	Laborer	9 (16.7%)	16 (29.6%)	29 (53.7%)	54 (100%)	
Socio-economic status	Upper	3 (12.0%)	10 (40.0%)	12 (48.0%)	25 (100%)	8.904 (0.179)
	Upper-middle	22 (23.4%)	26 (27.7%)	46 (48.9%)	94 (100%)	
	Lower middle	39 (18.0%)	60 (27.6%)	118 (54.4%)	217 (100%)	
	Low	68 (17.5%)	143 (36.8%)	178 (45.8%)	389 (100%)	
Ethnicity	Scheduled castes	17 (21.5%)	29 (36.7%)	33 (41.8%)	79 (100%)	6.456 (0.374)
	Scheduled tribes	6 (35.3%)	5 (29.4%)	6 (35.3%)	17 (100%)	
	Other backward castes	93 (17.7%)	174 (33.1%)	259 (49.2%)	526 (100%)	
	General	16 (15.5%)	31 (30.1%)	56 (54.4%)	103 (100%)	

* *Statistically significant at alpha = 0.05 level*.

The overall prevalence of current smoking was 11.7% (*n* = 85) and consumption of alcohol (at least once a week) was 5% (*n* = 36). However, 72.1% (*n* = 523) used any one form of smokeless tobacco daily. The summary of bivariate and regression analyses of the known risk factors for multimorbidity are given below in Table 2. The details of the regression models run are provided in eMethods 1 (Supplementary Material).

**Table 2:** Summary findings of bivariate and regression analyses of association between multimorbidity and some of its risk factors.

**Risk factor** **(ref** **=** **No)**		**Multimorbidity** ***n* (%)**		**X^**2**^** **(*p*-value)**	**Age-sex Adjusted OR** **(95% CL)**
		**Present**	**Absent**		
Current smoking	No	34 (40%)	51 (60%)	3.003 (0.083)	1.349 (0.806–2.257)
	Yes	320 (50%)	320 (50%)		
Smokeless tobacco	No	245 (46.8%)	278 (53.2%)	2.953 (0.086)	1.298 (0.924–1.822)
	Yes	109 (54%)	93 (46%)		
Alcohol consumption	No	11 (30.6%)	25 (69.4%)	5.062 (0.024)*	1.975 (0.911–4.283)
	Yes	343 (49.8%)	346 (50.2%)		
Family history of diabetes	No	266 (45.3%)	321 (54.7%)	15.227 (<0.001)*	1.67 (1.11–2.52)*
	Yes	88 (63.8%)	50 (36.2%)		
Family history of hypertension	No	193 (42.4%)	262 (57.6%)	20.091 (<0.001)*	1.80 (1.29–2.50)*
	Yes	161 (59.6%)	109 (40.4%)		

* *Statistically significant at alpha = 0.05 level*.

**Figure 2:**
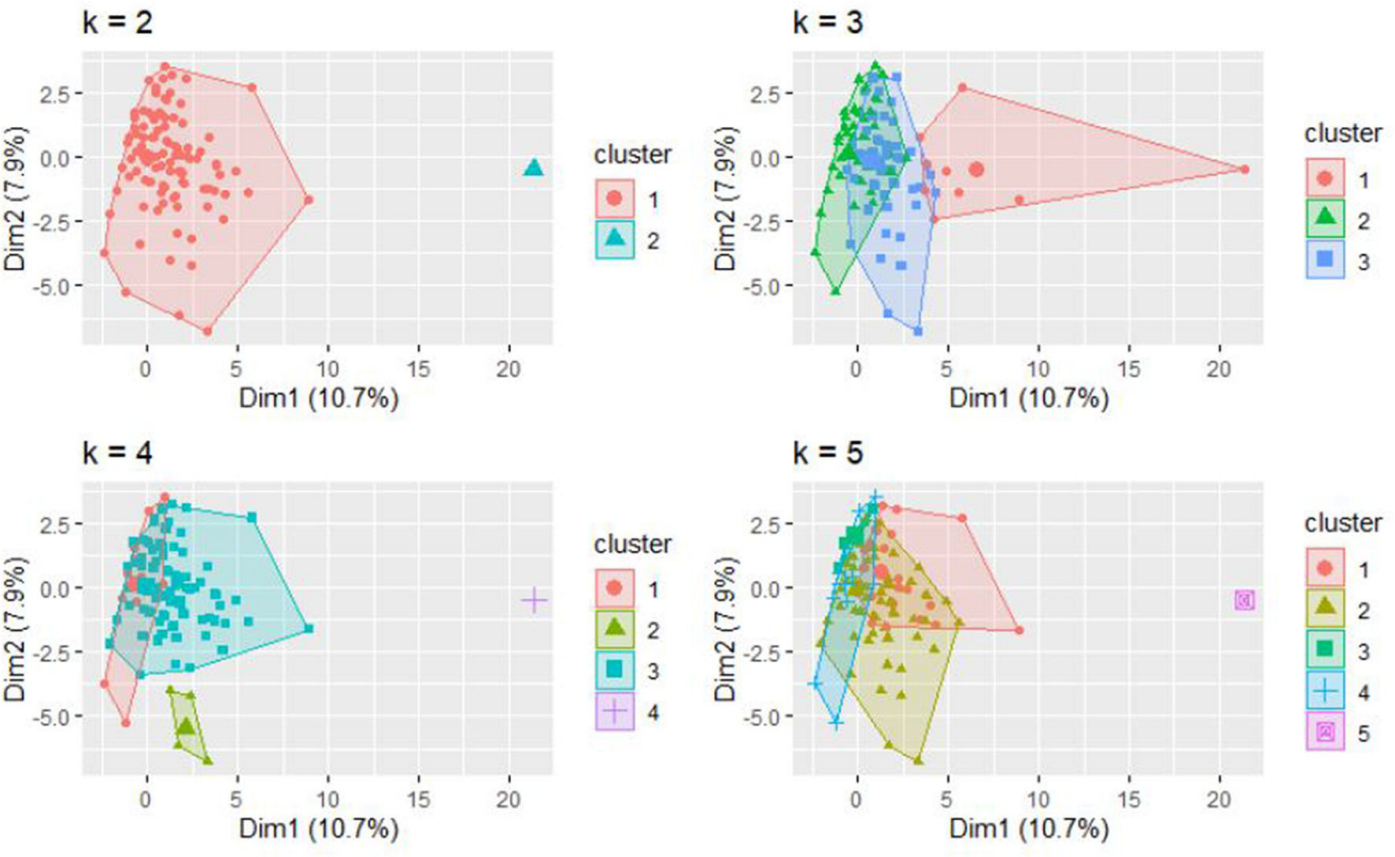
Two, three, four, and five (k=) clusters of observations based on K means clustering.

**Figure 3:**
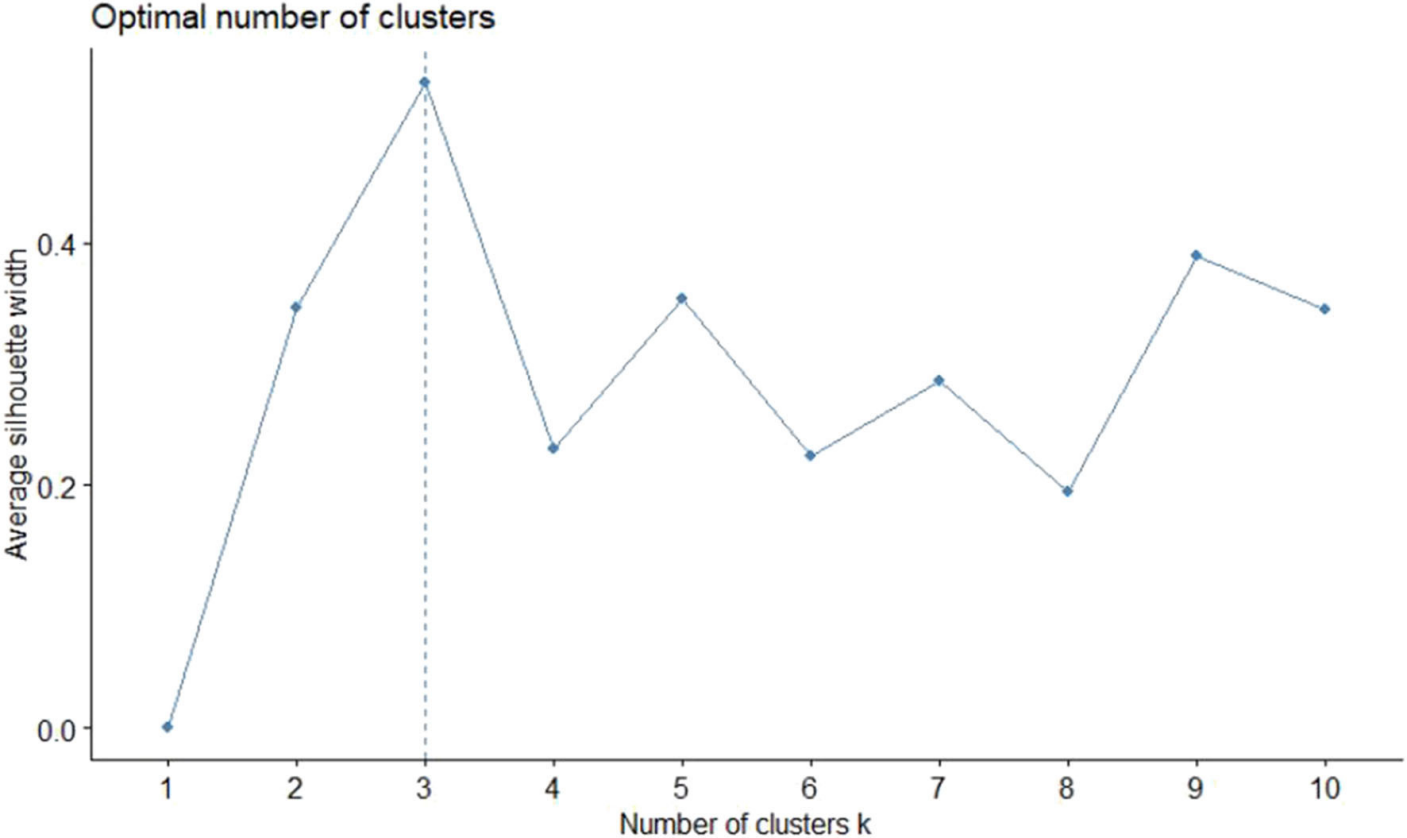
Optimal number of clusters estimated using average silhouette method.

**Figure 4:**
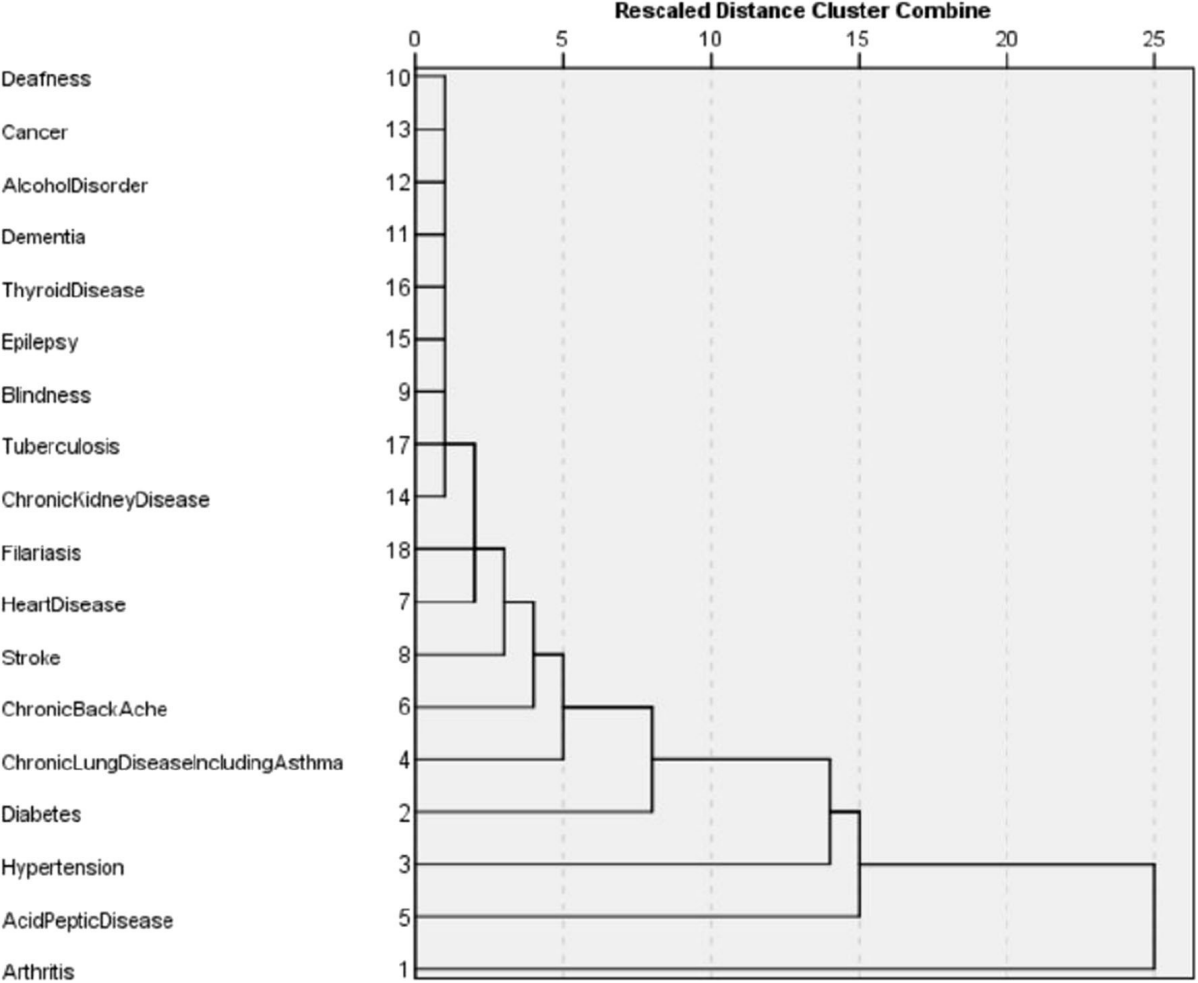
Cluster dendrogram for multimorbidity in males.

**Figure 5:**
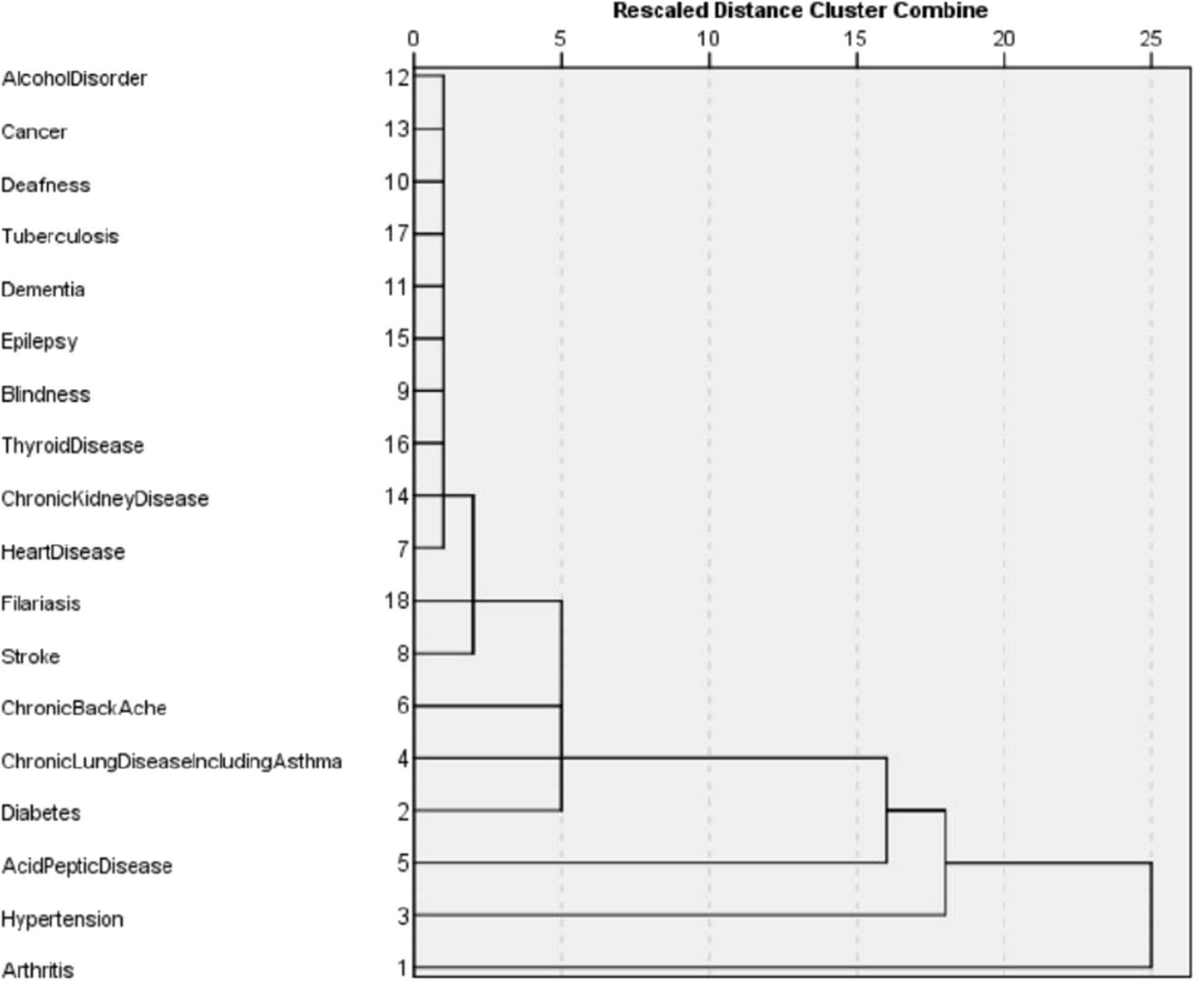
Cluster dendrogram for multimorbidity in females.

The dissimilarity proximity matrix of Euclidean distance was built and is provided in Supplementary Table 1. K-means clustering algorithm was run and the members of clusters were identified as given in Table 3 and Figure 2. Arthritis was a distinct cluster in itself for most iteration and so were Hypertension and Acid peptic disease when the cluster numbers were increased. The optimum number of clusters was 3 as given in Figure 3. The gender segregated dendrograms are given in Figures 4, 5.

**Table 3:** Membership of 2, 3, 4, and 5 clusters.

**Condition**	**Cluster membership**			
	**5 clusters**	**4 clusters**	**3 clusters**	**2 clusters**
Arthritis	1	1	1	1
Diabetes	2	2	2	2
Hypertension	3	3	3	2
Chronic lung disease including asthma	4	2	2	2
Acid peptic disease	5	4	3	2
Chronic backache	4	2	2	2
Heart disease	4	2	2	2
Stroke	4	2	2	2
Blindness	4	2	2	2
Deafness	4	2	2	2
Dementia	4	2	2	2
Alcohol disorder	4	2	2	2
Cancer	4	2	2	2
Chronic kidney disease	4	2	2	2
Epilepsy	4	2	2	2
Thyroid disease	4	2	2	2
Tuberculosis	4	2	2	2
Filariasis	4	2	2	2

## Discussion

The study was done among 725 rural older adult participants aged 60–106 years. While 80% of participants had chronic diseases, 48.8% had multimorbidity. Among the multimorbidity forms, dyads (two chronic conditions) were the most common (25%), followed by triads (15.2%). 8.7% participants had four or more chronic diseases. Analysis of “WHO-SAGE 2007” data had shown that 28.5% of the population was suffering from one chronic disease and 8.9% from multiple morbidities in India (18). In another study, the prevalence of multimorbidity in India was 23.6%, Kerala being the most affected state with prevalence of 42.02% followed by Punjab (35.78%) (8). In a systematic review by Pati et al., the prevalence of multimorbidity in South Asia ranged from 24.1 to 83% (3). In another integrative review by de Melo et al., the prevalence of multimorbidity among older adults ranged from 30.7 to 57% (19). A wide variation in the prevalence of multimorbidity has been observed depending on the study area and population. While a prevalence of 90.5% has been reported among Chinese adults, it was 44.05% among rural poor of Sundarban, West Bengal and 30.7% among older adults from some selected Indian states (7, 20, 21).

A study conducted by Verma and Mishra among North Indian older adults in 2019, reported that 33% had single chronic disease, 31.8% had dyads, 15.5% had triads, and 5.8% had four and more chronic diseases (9). Studies in our study region have shown the prevalence of multimorbidity to be 28.3% (10).

The prevalence of multimorbidity was slightly higher in females (50.4%) than males (47.4%) in our study. Similar female preponderance of multimorbidity has been reported by other studies as well (8–10, 22). However, according to a study by Mini et al., males had 1.5 times higher chance to develop multimorbidity (7). Gender predisposition might be a function of age and the prevalence of other specific diseases seen in a particular sex.

In our study, we found no significant difference between the mean age of the participants with and without multimorbidity. A study from North India in similar age groups from India has reported similar findings (9). In contrast, other studies have found that the older age groups to havea 1.9–2.8 times higher chance of developing multimorbidity compared to those between 60 and 69 years (7, 8). Similar increasing trends of multimorbidity prevalence with age are seen among adults as well in our study. A study by Pati et al. among adults attending primary care in the same region has shown that the number of chronic diseases to increase with increasing age (10). Similarly, other studies have shown a very steep rise in the prevalence of multimorbidity in the older adults when compared to young adults (21, 22). The relationship of multimorbidity with age among the older adults, particularly in rural settings does not seem similar to that in younger adults and needs further.

This study does not show any significant association between socio-economic status and multimorbidity. Other studies have found that the middle socio-economic group had a significantly higher prevalence of multimorbidity (63.6%) followed by lower class (57.1%) and upper class (41.3%) (9, 22). According by a few other studies, in comparison to lower SES, the middle and higher socio-economic group have 2–2.1 times and 3.9–4.6 times higher chance to develop multimorbidity, respectively (7, 8). In another study it was found that those with a deficit of wealth were 1.3–1.6 times more likely to develop multimorbidity (21). However, our study is based in rural population with a large proportion of the study participants are from the lower economic strata, which might affect the association.

Behavioral factors such as tobacco use (both smoking and smokeless tobacco) and alcohol consumption doesn't show any significant relation with multimorbidity in this study. While studies in rural Indians have shown similar results, other studies have found that those who were using tobacco and alcohol had a 1.2 times and 1.5 times higher chance of developing multimorbidity than those who were not (7, 21). Patients reported behavioral risk factors are dependent on the operational definitions used and other types of cultural biases in reporting known harmful behaviors which is an inherent limitation for observational studies and might influence the interpretations.

We have performed cluster analysis to classify clusters of chronic diseases. Cluster analysis starts by choosing the variables based on theory and previous literature. Then the dissimilarity (or similarity) between variables is measured using a distance and proximity matrix. We have used squared Euclidian distance for the measure of proximity. In the next step, the clusters are formed using different hierarchical or non-hierarchical algorithms. Here we use K-means clustering to establish the membership of clusters. This is followed by deciding on the optimum number of clusters using various instruments such as agglomerative schedule, elbow method, and gap method. We used the silhouette method to determine the optimum number of clusters (23, 24). Finally, graphical representations are built for the clusters. We used cluster plots and dendrograms. Cluster plots show the proximity of variables in a 2 dimensional Euclidean space and Dendrograms show the distance at which the clusters are combined (23, 25, 26).

In our study, the optimal number of clusters (in both males and females) was found to be 3. While Arthritis alone is placed in a cluster, hypertension, and acid peptic disease are in another cluster and in the last cluster all the rest conditions were placed. Different sets of chronic disease clusters have been reported from studies done in the United Kingdom (3 clusters), Brazil (3 clusters), Switzerland (4 clusters), USA (4 clusters), Portugal (6 clusters), and Australia (6 clusters) using separate methods of cluster analysis such as hierarchical agglomeration, correlation matrix with Yule's Q and exploratory factor analysis (27–32). However, the cluster membership is variable and depends on both the nature of the study population and statistical methods used in analysis. While interpretations of clustering of chronic diseases might have a role in formulating complementary diagnosis and management strategies in patient groups, clinical and pathophysiological correlations seem to play important roles as well. The findings presented in the study will have applications in improving clinical diagnoses algorithms, health service resource optimization and even AI logic development.

## Conclusion

To our knowledge, this is the first ever study done on multimorbidity among the rural older adult population in eastern India. Prevalence rate of 80% for chronic diseases and 48.8% for multimorbidity among rural older adults suggests the number of cases presenting at health facilities is only the tip of the iceberg. This warrants the urgent need of developing active surveillance strategies for policy makers and health program officers to early identify and promptly manage such cases to prevent their disability and improve their productivity. The cluster analysis using squared Euclidian distance for the measure of proximity suggests arthritis, hypertension, and acid peptic disease are the diseases that need to be prioritized and targeted for among the rural older adults.

## Data Availability Statement

The raw data supporting the conclusions of this article will be made available by the authors, without undue reservation.

## Ethics Statement

The studies involving human participants were reviewed and approved by Institutional human ethical committee of ICMR RMRC Bhubaneswar. The patients/participants provided their written informed consent to participate in this study.

## Author Contributions

JK conceptualized the study along with SP. TB and SB helped in data collection and analysis. Analysis and manuscript preparation was done by JK and TB. SB and SKP reviewed the manuscript and prepared the final draft. All authors contributed to the article and approved the submitted version.

## Conflict of Interest

The authors declare that the research was conducted in the absence of any commercial or financial relationships that could be construed as a potential conflict of interest.
