# Supplementary material for: Prevalence and Patterns of Multimorbidity Among Rural Elderly: Findings of the AHSETS Study
Source: Front Public Health. 2020 Nov 5;8:582663. doi: 10.3389/fpubh.2020.582663 (PMC7676903; doi:10.3389/fpubh.2020.582663)
Supplement: Supplementary file 1 [file Data_Sheet_1.PDF]

List of online only supplemental materials

1. eMethod-1. Regression Models used.
2. eTable-4. Euclidean distances proximity matrix of chronic conditions.

MATERIALS

eMethod-1. Regression Models used

Model-1

Log (π Multimorbidity /1-π Multimorbidity) = β 0 + β 1 (Current Smoking) + β 2 (Smokeless Tobacco) + β 3 (Alcohol consumption) + β 4 (Family history of Diabetes) + β 5 (Family history of Hypertension)

Where, (π Multimorbidity /1-π Multimorbidity) = Risk of Multimorbidity (Multimorbidity, No Multimorbidity) & amp; β 0 - β 5 = Regression Coefficients

Model-2

Log (π Multimorbidity /1-π Multimorbidity) = β 0 + β 1 (Current Smoking) + β 2 (Smokeless Tobacco) + β 3 (Alcohol consumption) + β 4 (Family history of Diabetes) + β 5 (Family history of Hypertension) + β 6 (Age) + β 7 (Male Gender)

Where, (π Multimorbidity /1-π Multimorbidity) = Risk of Multimorbidity (Multimorbidity, No Multimorbidity) & amp; β 0 - β 7 = Regression Coefficients

eTable-4. Euclidean distances proximity matrix of chronic conditions

Chronic condition	Arthritis	Diabetes	Hypertension	Chronic Lung Disease Including Asthma	Acid Peptic Disease	Chronic BackAche	Heart Disease	Stroke	Blindness	Deafness	Dementia	Alcohol Disorder	Cancer	Chronic Kidney Disease	Epilepsy	Thyroid Disease	Tuberculosis	Filariasis
Arthritis	.000	1.000	.859	.956	.774	.922	.978	.978	1.000	.990	.995	.988	.993	.976	.990	.993	.983	.983
Diabetes	1.000	.000	.536	.313	.646	.313	.228	.238	.221	.211	.211	.214	.214	.221	.221	.204	.223	.252
Hypertension	.859	.536	.000	.614	.650	.619	.612	.563	.619	.609	.614	.607	.607	.595	.609	.602	.626	.631
Chronic Lung Disease Including Asthma	.956	.313	.614	.000	.583	.245	.170	.209	.167	.158	.163	.155	.160	.167	.167	.170	.170	.199
Acid Peptic Disease	.774	.646	.650	.583	.000	.568	.585	.600	.583	.578	.583	.580	.580	.583	.578	.585	.585	.595
Chronic Back Ache	.922	.313	.619	.245	.568	.000	.165	.194	.143	.133	.138	.131	.136	.148	.138	.146	.146	.165
Heart Disease	.978	.228	.612	.170	.585	.165	.000	.090	.044	.034	.039	.032	.036	.049	.044	.041	.046	.080

Stroke	.978	.238	.563	.209	.600	.194	.090	.000	.073	.063	.068	.061	.066	.073	.073	.075	.080	.109
Blindness	1.000	.221	.619	.167	.583	.143	.044	.073	.000	.007	.012	.010	.010	.032	.017	.019	.024	.053
Deafness	.990	.211	.609	.158	.578	.133	.034	.063	.007	.000	.002	.000	.000	.022	.007	.010	.015	.044
Dementia	.995	.211	.614	.163	.583	.138	.039	.068	.012	.002	.000	.005	.005	.027	.012	.015	.019	.044
Alcohol Disorder	.988	.214	.607	.155	.580	.131	.032	.061	.010	.000	.005	.000	.002	.019	.010	.012	.017	.046
Cancer	.993	.214	.607	.160	.580	.136	.036	.066	.010	.000	.005	.002	.000	.024	.010	.012	.017	.046
Chronic Kidney Disease	.976	.221	.595	.167	.583	.148	.049	.073	.032	.022	.027	.019	.024	.000	.032	.034	.039	.068
Epilepsy	.990	.221	.609	.167	.578	.138	.044	.073	.017	.007	.012	.010	.010	.032	.000	.019	.024	.053
Thyroid Disease	.993	.204	.602	.170	.585	.146	.041	.075	.019	.010	.015	.012	.012	.034	.019	.000	.027	.056
Tuberculosis	.983	.223	.626	.170	.585	.146	.046	.080	.024	.015	.019	.017	.017	.039	.024	.027	.000	.061
Filariasis	.983	.252	.631	.199	.595	.165	.080	.109	.053	.044	.044	.046	.046	.068	.053	.056	.061	.000
